# Supplementary material for: Synergistic morphology and feedback control for traversal of unknown compliant obstacles with aerial robots
Source: Nat Commun. 2024 Mar 26;15:2646. doi: 10.1038/s41467-024-46967-5 (PMC10965961; doi:10.1038/s41467-024-46967-5)
Supplement: Supplementary file 1 — Supplementary Information [file 41467_2024_46967_MOESM1_ESM.pdf]

SUPPLEMENTARY INFORMATION

Synergistic Morphology and Feedback Control for Traversal of Unknown Compliant Obstacles with Aerial Robots

Authors and Affiliations:
Emanuele Aucone[1,2,*], Christian Geckeler[1,2], Daniele Morra[3], Lucia Pallottino[3], and Stefano Mintchev[1,2]

[*] Corresponding author. Email: eaucone@ethz.ch
[1] Environmental Robotics Laboratory, Department of Environmental Systems Science, ETH Zürich, Zürich, Switzerland.
[2] Swiss Federal Institute for Forest, Snow and Landscape Research, WSL, Birmensdorf, Switzerland.
[3] Research Center "E. Piaggio", Department of Information Engineering, University of Pisa, Pisa, Italy.

This files contains:

Supplementary Method 1: Drone Architecture
Supplementary Method 2: Compliant Hinged Plate
Supplementary Method 3: Controller Implementation
Supplementary Method 4: Runtime Execution
Supplementary Method 5: Stability and Feasibility of the NMPC-based Controller
Supplementary Method 6: Impact of NMPC Objective Cost Weights
Supplementary Method 7: Multiple Obstacles Traversal
Supplementary Discussion 1: Sim-to-Real Gaps
Supplementary References
Supplementary Table 1: Inertial parameters of the designed aerial robot
Supplementary Table 2: Linear and torsional stiffness of the rotating joint during the experiments
Supplementary Table 3: Average value for the 5 metrics used to compare the performances in simulation and real-world experiments
Supplementary Figure 1: Hinged surface used during the experiments
Supplementary Figure 2: Simulation tools exploited to validate the stability and feasibility of the controller, and to evaluate its performance in terms of optimization
Supplementary Figure 3: Ablation study to evaluate the impact of the cost weights on the controller performances
Supplementary Figure 4: Traversal of multiple obstacles
Supplementary Figure 5: Assessment of the sim-to-real gaps for experiments conducted with balanced weight

Supplementary Method 1: Drone Architecture

The quadrotor is composed of a carbon-fiber frame, DYS Thor 2408-2200KV brushless motors equipped with 6-inch 2-blade propellers (each providing about 8 N lift force), a BetaFlight Flight Controller (FC) for attitude control, and a Khadas VIM3 companion computer for high-level control, communication, and additional onboard computations. A tracking camera Intel Realsense T261 provides onboard odometry measurements. To measure the external wrench acting on the drone's CoM, we use a 6-axis Force/Torque sensor (Medusa, Bota Systems AG) that connects the quadrotor frame with the frame of the protective shell. The system is powered with a 3-cell 2500 mAh LiPo battery and has a total mass of 1.1 kg. This value has been measured with an accurate scale, and it differs from the one computed by the CAD, which can happen due to mechanical inaccuracies associated with the manufacturing process, as well as additional modelling inaccuracies (e.g. cabling). The moments of inertia of the real system have been estimated by linearly scaling the inertias output from the CAD, using the ratio between the real mass and the one from the CAD as scaling factor. This assumes that the extra mass is uniformly distributed on all the components, without a change in the dimension, therefore linearly affecting the moments of inertia. It is worth noticing that high model inaccuracies may generally degrade the performance of model-based controllers. Inertial parameters useful for the dynamical model inside the NMPC are reported in Supplementary Table 1.

Supplementary Method 2: Compliant Hinged Plate

The hinged surface used during the experimental validation is a lightweight construction foam plate, with a weight of around 400 g, and dimensions of 60 cm by 80 cm by 2 cm. The plate is attached to a fixed structure via two hinges, which allow the foam plate to rotate around one axis (vertical one), forming a single rotational joint (Supplementary Fig. 1A). Each of the hinges has a rotating part connected to a linear spring with stiffness k_{spring} that gives the obstacle a compliant behavior, as it returns the surface to its initial position and provides the force which the quadrotor has to counteract (Supplementary Fig. 1B). In detail, since the springs are in parallel, the linear stiffness of the rotational joint is the sum of the two contributions $k_{linear} = k_{spring1} + k_{spring2}$. By fixing the radius of the hinge (in our case $R_{hinge} = 6mm$), the equivalent torsional stiffness $k_{torsional}$ of such rotational joint (Supplementary Fig. 1C) can be changed by changing the linear stiffness k_{linear} (thus the springs themselves), according to the following relation: $k_{torsional} = k_{linear} * R_{hinge}^2$. For our experiments, the effective torsional stiffness values range across 1 order of magnitude (Supplementary Table 2), with initial point of contact located about 60 cm from the axis of the hinge.

Supplementary Method 3: Controller Implementation

We developed and validated the NMPC and the overall strategy using the Robot Operating System (ROS) and Gazebo 3D simulator (Supplementary Fig. 2A). We used the RotorS[1] package for simulating a quadrotor, as well as its dynamics and flight architecture; then, we incorporated a F/T sensor with a plugin that simulates measured with Gaussian noise; finally, we included the 3D model of the cage exported from the CAD – the visual model of the cage is a sphere but the interaction occurs only on the ring and the inertial parameters corresponds to the ones used for the real-world prototype (Supplementary Table 1). We used a Gazebo plugin to generate an elastic behavior of the environment, by using one rotational spring joint. We performed several experiments in the 3D physical simulator for implementation, debug, tuning and evaluation. A study on the sim-to-real gaps is reported in a dedicated section (see Supplementary Sec. Sim-to-Real Gaps). For the real drone, we exploited the RPG Quadrotor Control package[2] for autopilot functionalities, and then implemented our NMPC previously developed in simulation. All the components were executed on the onboard companion computer during the experiments. We used Ubuntu 18.04 and ROS Melodic for sensor data acquisition (both tracking camera and F/T sensor), for the controller computation, for connection and communication with the Flight Controller, and for online data logging.

Supplementary Method 4: Runtime Execution

We chose a prediction horizon t = 1 second, sampled at N = 20 steps/nodes. Therefore, the discretization time of the NMPC algorithm, i.e. the length of one of the N steps, results in 0.05 seconds. The internal MPC prediction is hence performed at 20 Hz, whereas the controller runs at a frequency of 40 Hz. On

average, the computation time needed for the algorithm to solve the OCP was 0.018 seconds.

At runtime, for each iteration, the NMPC receives the data from the sensors (i.e. the external wrench and the state), sets the initial state condition using the current values of the state, and integrates the continuous dynamics in order to discretize it. Eventual inaccuracies in the sensor readings may directly affect the evolution of the dynamics, so it is important to be sure that the sensors used onboard are accurate enough to allow to find a feasible solution of the NMPC. In our approach, the only physical parameters required for the dynamics of the system used in the optimization are the inertial properties of the drone (mass and moments of inertia, Supplementary Table 1). Generally, inaccuracy in the mass just results in a difference of the thrust force that the drone has to generate, whereas mismatches in the inertia, thus on the rotational dynamics, may result in degraded performances of the controller. Since the drone operates in quasi-static motion and the main contribution in the rotational dynamics (last row of Eq. 1) comes from the external torques, variations on the moments of inertia have a small impact during the interaction. We experimentally validate this and report the results in Supplementary Sec. Sim-to-Real Gaps. Then, the algorithm gets the upper and lower bounds for the constraints, as well as the objective weights, and computes the cost vectors. Thus, it starts the optimization from a virtual hovering condition (at the first initialization) or from the previous NMPC solution (for the following iterations); once the optimization converges, the NMPC returns both control $\boldsymbol{u}$ and state variables $\boldsymbol{x}$ along the whole prediction horizon. From the control vector $\boldsymbol{u}$, the collective thrust at step 0 is output as c_{cmd}. Due to the hardware architecture of our platform (underactuated), we output a commanded orientation for the downstream low-level attitude controller, instead of the torque τ at step 0 from vector $\boldsymbol{u}$. This orientation command is in the form of a quaternion $\boldsymbol{q}_{cmd}$, and it refers to the orientation computed by the NMPC, as part of the state $\boldsymbol{x}$, at step 1 of the optimization horizon, i.e. the expected orientation computed by the optimization at step 1 in response to the applied torque at step 0. Despite the good performance demonstrated during the experiments, outputting the 'suboptimal' quaternion, instead of the optimal torques, results in a mismatch between the predicted NMPC dynamics and the actual one coming from the downstream system. This might potentially lead to sluggish or unwanted behaviors, which were avoided thanks to the physical constraints imposed in the optimization and the quasi-static motion condition.

Furthermore, the OCP can incorporate the evolution of an external wrench in the receding horizon. As common practice, we choose to keep the wrench constant (zero derivative) during the prediction (1 second). Nevertheless, the NMPC internally updates the external wrench with a new value every 0.05 seconds and performs the optimization again. Without loss of generality, this assumption is thus justifiable i) because the reference velocity we target is really small (-0.15 m s^{-1}), making the quadrotor motion quasi-static, and ii) because the average variation between two updates of external wrench is quite limited throughout the execution of the task (variation of longitudinal force within 0.05 seconds is on average around 0.02 N). To further avoid jumps in the wrench measures, and consequent bouncing effects, we add a filter in the loop to smoothen the external wrench received by the NMPC. This guarantees to avoid abrupt increases and decreases of the wrench, which is beneficial especially during first contact and detachment (i.e., when moving from a zero to non-zero wrench). The filter further removes eventual biases by exploiting measures during the hovering condition. The usage of such approach makes the overall system less sensitive to inaccuracies in the direct wrench measurements. It is worth to mention that this might be a limitation in scenarios with a rapidly varying external wrench or when the quasi-static motion is not guaranteed, potentially affecting the stability of the system or the performance of the interaction.

Supplementary Method 5: Stability and Feasibility of the NMPC-based Controller

The stability and feasibility of receding horizon problems are generally not guaranteed except in cases of infinite horizon control. If the prediction horizon is limited to N steps, stability and feasibility are questionable. Thus, we opted for a finite receding horizon for our approach. In principle, it has been shown that under the assumption that the reference trajectory is consistent with the vehicle dynamics, the stability and feasibility is guaranteed by selecting a sufficiently large prediction horizon length[3], at the cost of a higher computation effort. Further, terminal cost and terminal constraints can be chosen such that closed-loop stability and feasibility are ensured[4]. For applications on aerial robot, the computation power is often limited by the platform, yet fast control action are needed, so it is important to

find a proper balance between the two aspects.

For our work, we decided for use a terminal cost, as it is easy to compute and its weights can be directly tuned, whereas terminal constraints are generally more difficult to solve [5]. The approach has been validated in several work, e.g. for path following [6], where the authors provided closed-loop asymptotic stability of MPC without stabilizing constraints, as well as for physical interaction with a UAV [7]. Since our problem relates to path following, the reference trajectory is defined by the forward velocity and the position in the other two axes, which is feasible with respect to the limits and the dynamics of the system. By combining a low reference velocity (quasi-static motion) and the constraints on the orientation (which act as an additional safety margin), we ensure that the drone did not have aggressive behavior and the controller could find a feasible solution – i.e. the NMPC has no hard constraints, only limits on the angles and thrust, so the OCP is even easier to solve, as it just derives how the external wrench affects the dynamics and what is the desired impedance acceleration in accordance to the external stimuli, while keeping track of following the path in parallel. To quantitively evaluate the stability and feasibility, we performed several simulations with different values of horizon length, achieving a trade-off between stability performance and computational load. In detail, we tuned the horizon length N (steps) by gradually increasing it, finding a value high enough to allow to always find a feasible and stable solution and small enough not to increase the computation time. In parallel, we monitored in simulation the distance between the predicted trajectory over the horizon and the executed trajectory (Supplementary Fig. 2B), as well as the value of the cost function, to be sure that the NMPC could effectively find an admissible solution at all the iterations. This metric helps to check for eventual problems in both the NMPC implementation (to validate that the model and the constraints are feasible and properly defined) and the solver algorithm. Finally, it is worth noticing that the feasibility and stability of the NMPC solution in our approach are further simplified since our strategy does not use switching conditions between free-flight and contact, as well as between the interaction modes – switching conditions in the optimization problem may create discontinuities in the solution, which are generally mitigated with high-level logics to allow smooth transitions [8].

Supplementary Method 6: Impact of NMPC Objective Cost Weights

To quantitatively assess the impact of the controller parameters on the performance of the system in terms of path following and safe physical interaction – the two main objectives of the NMPC – we perform an ablation study in simulation for different weight parameters included in the optimization cost vectors (Supplementary Fig. 3 and Supplementary Movie S5). Variability among the tests is ensured using simulated noise on the F/T sensor, as the controller will receive different sensor readings during each experiment and consequently have a different behavior depending on the interaction forces.

We use 5 metrics related to the tracking of the lateral position and the forward velocity, as well as the stability of the drone during the interaction, in terms of applied force and attitude oscillations (on both roll and pitch) – see section Statistical Analysis for details about the metrics. We defined 3 sets of parameters: i) ***High Traj, Low Imp*** – weights related to the path following higher (at least 2 orders of magnitude) with respect to the weights related to the interaction (impedance), ii) ***Mid Traj, Mid Imp*** – balanced weights (same order of magnitude or 1 order different), iii) ***Low Traj, High Imp*** – weights related to the path following lower (at least 2 orders of magnitude) with respect to the weights related to the interaction. We performed several experiments for each set and for each value of stiffness (low, mid, high), to calculate the success rate, and conducted a statistical analysis on a subset of 5 experiments to assess the impact of the weight parameters on the performance of the system.

As depicted in the Supplementary Fig. 3, when the path following has a higher weight in the optimization (***High Traj, Low Imp)***, the errors in the lateral position are smaller (Suppl. Fig. 3A). However, during contact the drone interacts more aggressively, due to the penalization of the impedance term, and sometimes cannot safely dampen the elastic behavior of the environment. The unwanted behavior during the interaction can be seen by higher values of longitudinal velocity error (Suppl. Fig. 3B), higher longitudinal force (Suppl. Fig. 3C), as well as higher oscillations on the attitude (Suppl. Fig. 3D and E). This effect increases when the obstacle gets stiffer, as it has a stronger response upon interaction. In this scenario, the success rate was 100%, 80% and 40% for low, mid, and high stiffness respectively (Suppl. Fig. 3F).

Viceversa, when the impedance weights have a higher impact with respect to the path following ones (***Low Traj, High Imp***), the stability greatly improves (Suppl. Fig. 3C, D, E) but the performances in tracking the reference path degrade; the errors in the lateral position, for each stiffness, are the highest among the three sets, and almost reach 0.5m in magnitude (Suppl. Fig. 3A). Success rate was 100% for all the stiffness values. Balancing the weights for the two objectives (***Mid Traj, Mid Imp***) still allows to ensure a safe behavior during the interaction (Suppl. Fig. 3C and D) and to reduce the errors in tracking the reference path (with respect to the previous set of weights – Suppl. Fig. 3A and B), finding a good compromise between the two objectives.

Supplementary Method 7: Multiple Obstacles Traversal

We have performed experiments in our simulation environment to investigate the behavior of the system during interaction with multiple obstacles (Supplementary Fig. 4 and Supplementary Movie S6). This is an initial investigation to draw insights into future developments.

During contact with multiple obstacles, the force/torque sensor only measures the net wrench acting on the drone. Due to the implementation of the strategy, the controller is consequently informed with a single wrench. We have experimentally tested that the drone might be able to traverse the obstruction in some cases, or fail, depending on the stiffness and location of the obstacles with respect to the drone itself and to each other.

First, we investigated a scenario where the drone (and the path to follow) is centered with respect to two obstacles, aligned and spaced apart from each other (Supplementary Fig. 4A) – we tested for obstacles having either same or different stiffness. In this scenario, the drone enters in contact with both obstacles simultaneously and the net wrench resulting from the two contacts is informative enough to guide the drone towards the other side of the obstruction: for obstacles with equal stiffness, the drone simply proceeds straight along the path as the contribution of the lateral forces cancels out; during interaction with obstacles having different stiffness, instead, the lateral component of the resultant wrench will guide the drone towards the most compliant obstacle, as if the interaction would be with a single obstacle.

A second scenario involves two obstacles misaligned with respect to each other (Supplementary Fig. 4B). We noticed that the drone starts to interact with the first obstacles, but upon contact with the second one failure cases might happen for some misalignments and if the second obstacle is very stiff (almost rigid): indeed, once the drone pushes aside the first obstacle and enters in contact with the second, rigid one, it might get stuck due to the strong contribution of the second wrench on the net wrench, which is opposite to the path direction. This general scenario is likely to be encountered in natural environments, as obstacles have different compliance and are generally spaced in a very heterogeneous way. Thus, such insights highlights that future investigation would require to include decision-making capabilities onboard, mostly based on i) enhancing the perception system to detect and locate multiple contacts, as well as estimating the compliance of different obstacles, and ii) developing planning strategies that exploit such information to adapt the path online – e.g. using the information on the location of contacts and map the compliance of the environment to move towards more compliant areas.

Supplementary Discussion 1: Sim-to-Real Gaps

To quantitatively assess the gaps between our custom simulation environment and the real-world setup, we performed an analysis that compares the performances between simulated and real-world experiments in terms of the 5 metrics defined in the Methods chapter. For evaluation, the experiments with the set of balanced weights were used for both sim and real. To have the same number of samples in the distribution of data we extended the simulation tests. The statistical analysis is reported in Supplementary Fig. 5, whereas Supplementary Table 3 summarizes the study by considering the average value within the distribution of data, for each metric, to have a more comprehensive view of our findings.

In terms of interaction, the gap in the longitudinal forces is small (Supplementary Fig. 5C), with the highest discrepancy for low stiffness (Supplementary Table 3). This was expected as we simulated a F/T sensor with Gaussian noise, yet the load cell mounted on the real drone might have different types of additive noise and non-static biases that drift over time. Another reason that creates a gap relates to the fact that on the real drone the sensor is affected by strong vibrations due to the attachment with

the rigid frame of the drone, as well as by aerodynamic effects of the airflow on the cage, which is placed right below the propellers. Such effect, which is missing in simulation, introduces additional disturbance on the sensor readings. It is worth mentioning that in our study contact force models are not included in the NMPC prediction model, since the interaction is with an unknown environment (as discussed in the Suppl. Info.). The interaction, indeed, relies on the impedance term that directly exploits the measure of the sensor. This means that both in sim and real-world experience, despite the noise might be different, the impedance term allows the NMPC to operate under uncertainties by using a reactive behavior (as discussed in the Suppl. Info. and in[9]).

In terms of tracking performance, instead, the effect of having state estimator between simulation and reality is more evident in the results, despite small gaps. The real drone, indeed, uses a tracking camera running VIO onboard – the signals from this sensor are less accurate than the simulated ground truth. Generally, the consequence of having a tracking camera (which can be affected by noise as well as drifts, especially in variables like velocity which is derived from position) is quite more evident on predictive controllers w.r.t. reactive ones (as in the previous case), given the fact that a wrong state estimation generates an erroneous evolution of the model internally simulated and, in turn, a misleading control input that finally produces an inaccurate trajectory tracking[10]. Supplementary Table 3 shows similar errors in terms of tracking the lateral position, which is expected as the drone needs to slightly drift from the reference path while sliding on it (more evident for increasing stiffness). In terms of variability of these errors, Supplementary Fig. 5A confirms that the ground truth of the simulation ensures a more repeatable behavior, against the high dispersion of data for real-world experiment, which is due to the inaccuracies of the state estimation itself. Tracking performance in terms of velocity errors are more visible in terms of average values (Supplementary Table 3) as well as statistical difference (Supplementary Fig. 5B, P value ¡ 0.01). The gap, however, is in the order of 0.01 m s^{-1}. Tracking performance can be enhanced by using a motion-capture system that provides estimation of position, velocity, and orientation of the drone with an accuracy below the millimeter.

The oscillations on the attitude (both roll and pitch) exhibit the highest difference between sim and real-world. The statistical analysis highlights a difference with a significance of 99% (Supplementary Fig. 5D, E), as the oscillations on the attitude are limited to values below 1 degree. Despite this gap, Supplementary Table 3 reminds that the oscillations experienced on the real drone are limited to a few degrees, lower than the safety limits imposed by the NMPC ([-20,20] deg). A less accurate state estimate for the real-world scenario leads to measurements that present some noise and a higher dispersion also in this case (Supplementary Fig. 5D, E), as the attitude is provided by the VIO. Further, we believe that the higher values of oscillations on the real drone also relate to inaccuracies on the inertia between simulated and real drone, which directly affects the rotational dynamics. Sensor-based controllers use instantaneous sensor measurement, instead of an explicit model, to represent system dynamics, thus they are more robust against model uncertainties and external disturbances. It is known instead that model uncertainties in the form of unmodeled complex aerodynamic effects, varying payloads and parameter mismatch can degrade the overall system performance for model-based controllers.

To conclude, the analysis on the sim-to-real gap has helped to identify gaps, which highlight that the simulation is more conservative and it better ensures the quasi-static motion, thanks to a higher accuraciy of the sensors used on-board, as well as a perfect modeling of the dynamics' parameters used by the NMPC. Our objective was not to have a high-fidelity simulator for perfect sim-to-real transfer, but rather to exploit it to validate the idea of the combination of body morphology and minimalistic controller for the task of traversing compliant obstacles. In our case, the gaps have been shown to be small enough to avoid a severe degradation of the performance of the system after the transfer from simulation. In the future, a first direction relates to estimating the real inertia of the drone, which can surely help to make the model-based controller more robust and achieve better performance. Further, to compensate for additional uncertainties and disturbances on the rotational dynamics, the uncertainties could be considered as a variable in the rotational dynamics' equations as a safety measure[11], they could be further estimated online to make the MPC adaptive[12], or a downstream controller could be added to regulate the original NMPC command[13]. These approaches have been validated for free-flight at high speed (not for interaction tasks), yet have proved to improve both robustness to mismatches and tracking performance.

Supplementary References

1. Furrer, F., Burri, M., Achtelik, M, & Siegwart, R. Rotors – a modular gazebo mav simulator framework. *Studies in Computational Intelligence* **625**, 595–625 (2016).

2. Faessler, M., Franchi, A., & Scaramuzza, D. Differential flatness of quadrotor dynamics subject to rotor drag for accurate tracking of high-speed trajectories. *IEEE Robotics and Automation Letters* **3**, 620–626 (2018).

3. Grüne, L., Pannek, J., Seehafer, M. & Worthmann, K. Analysis of unconstrained nonlinear mpc schemes with time varying control horizon. *SIAM Journal on Control and Optimization* **48**, 4938–4962 (2010).

4. Borrelli, F., Bemporad, A., & Morari, M. Predictive Control for Linear and Hybrid Systems. *SIAM Journal on Control and Optimization* (Cambridge University Press, Cambridge, 2017).

5. Mayne, D., Rawlings, J., Rao, C. & Scokaert, P. Constrained model predictive control: Stability and optimality. *Automatica* **36**, 789–814 (2000).

6. Mehrez, M. W., Worthmann, K., Mann, G. K. I., Gosine, R. G. & Faulwasser, T. Predictive path following of mobile robots without terminal stabilizing constraints. *IFAC-PapersOnLine* **50**, 9852–9857 (2017).

7. Kocer, B. B., Tjahjowidodo, T., & Seet, G. G. L. Model predictive uav-tool interaction control enhanced by external forces. *Mechatronics* **58**, 47-57 (2019).

8. Peric, L., Brunner, M., Bodie, K., Tognon, M. & Siegwart, R. Direct force and pose nmpc with multiple interaction modes for aerial push-and-slide operations. *2021 IEEE International Conference on Robotics and Automation (ICRA)* 131–137 (2021).

9. Alharbat, A., Esmaeeli, H., Bicego, D., Mersha, A. & Franchi, A. Three Fundamental Paradigms for Aerial Physical Interaction Using Nonlinear Model Predictive Control. *2022 International Conference On Unmanned Aircraft Systems (ICUAS).* pp. 39-48 (2022).

10. Bicego, D., Mazzetto, J., Carli, R., Farina, M., A. & Franchi, A. Nonlinear Model Predictive Control with Enhanced Actuator Model for Multi-Rotor Aerial Vehicles with Generic Designs. *Journal of Intelligent & Robotic Systems.* pp. 1213-1247 (2020).

11. Sun, S., Romero, A., Foehn, P., Kaufmann, E. & Scaramuzza, D. A Comparative Study of Nonlinear MPC and Differential-Flatness-Based Control for Quadrotor Agile Flight. *IEEE Transactions On Robotics.* **38**, 3357-3373 (2022).

12. Hanover, D., Foehn, P., Sun, S., Kaufmann, E. & Scaramuzza, D. Performance, Precision, and Payloads: Adaptive Nonlinear MPC for Quadrotors. *IEEE Robotics And Automation Letters.* **7**, 690-697 (2022).

13. Nan, F., Sun, S., Foehn, P. & Scaramuzza, D. Nonlinear MPC for Quadrotor Fault-Tolerant Control. *IEEE Robotics And Automation Letters.* **7**, 5047-5054 (2022).

Suppementary Tables

**Inertial Parameters**	**Values**
Mass [kg]	1.100
Inertia along x-axis [kg m^2]	0.007
Inertia along y-axis [kg m^2]	0.007
Inertia along z-axis [kg m^2]	0.012

Supplementary Table 1: **Inertial parameters of the designed aerial robot.**

k_{linear} [N mm^{-1}]	$k_{torsional}$ [N mm rad^{-1}]
0.5	18
2.16	77.76
4.32	155.52

Supplementary Table 2: **Linear and torsional stiffness of the rotating joint during the experiments.**

Metrics	Low stiffness		Mid stiffness		High stiffness	
	**Sim**	**Real**	**Sim**	**Real**	**Sim**	**Real**
Pos error [m]	0.051	0.0945	0.104	0.110	0.141	0.088
Vel error [m s^{-1}]	0.035	0.108	0.040	0.101	0.045	0.110
Max force [N]	0.700	1.292	1.154	1.394	1.592	1.719
Roll oscillations [deg]	0.174	2.973	0.303	2.497	0.443	3.010
Pitch oscillations [deg]	0.405	4.029	0.498	5.138	0.611	5.833

Supplementary Table 3: **Average value for the 5 metrics used to compare the performances in simulation and real-world experiments.**

Suppementary Figures

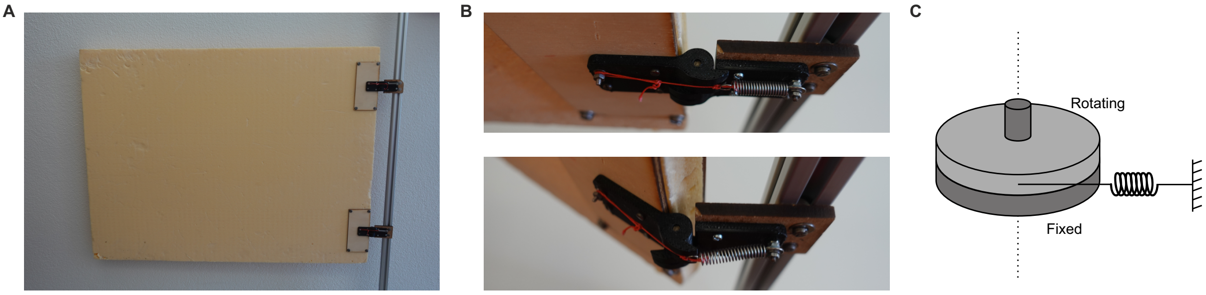

Supplementary Figure 1: **Hinged surface used during the experiments.** **A** Foam plate attached to a rigid pole via two hinges. **B** Single hinge in the resting condition and when the plate is deflected. **C** Diagram of a single hinge mechanism.

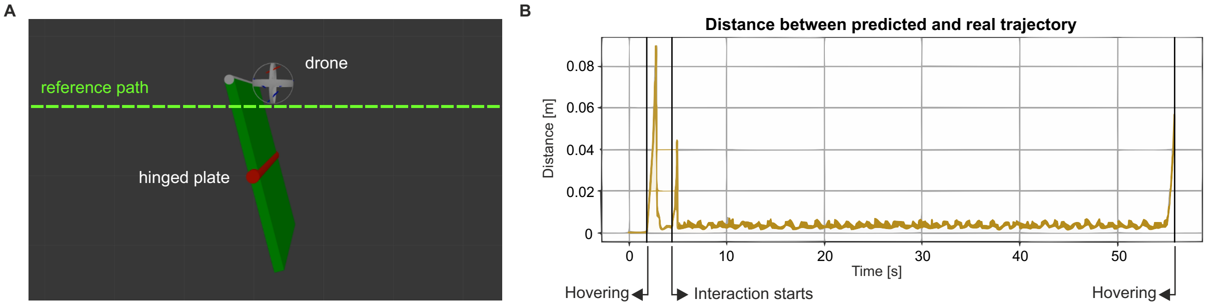

Supplementary Figure 2: **Simulation tools exploited to validate the stability and feasibility of the controller, and to evaluate its performance in terms of optimization.** **A** Gazebo 3D environment developed to test our solution. **B** Plot of the distance between predicted and real, executed trajectory over time, from an experiment where the parameters were properly tuned.

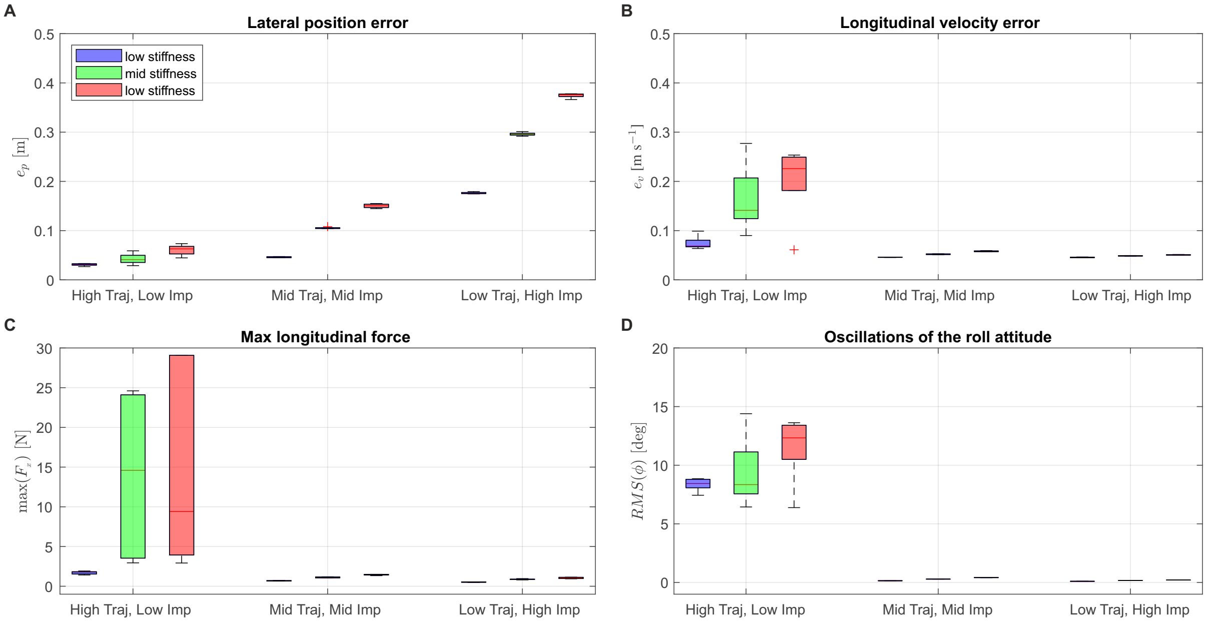

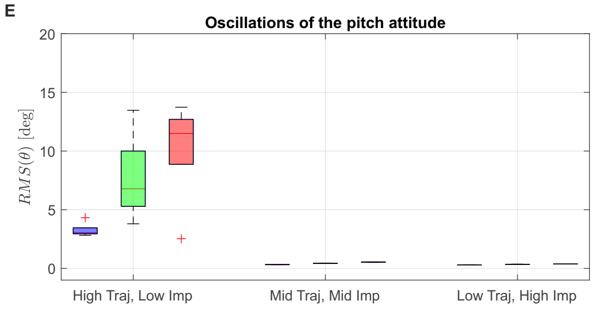

F

Success Rate (%)

Weights set	Low stiffness	Mid stiffness	High stiffness
High Traj, Low Imp	100	80	40
Mid Traj, Mid Imp	100	100	100
Low Traj, High Imp	100	100	100

Supplementary Figure 3: **Ablation study to evaluate the impact of the cost weights on the controller performances. A** Tracking error of the lateral position; **B** longitudinal velocity; **C** max longitudinal force; **D** oscillations on the roll motion; **E** oscillations on the pitch motion. Mann-Whitney U test performed among the three sets and stiffnesses; box plots indicate median (middle line), 25th, 75th percentile (box) and 5th and 95th percentile (whiskers) as well as outliers (single points); number of tests used for the statistical analysis = 5 for each stiffness and set of weights; **P $<$ 0.01 for all the boxplots compared among the sets, for each stiffness - asterisks not added to the plot for clarity of the figure. **F** Success Rate (%) for the different values of stiffness and different set of weights.

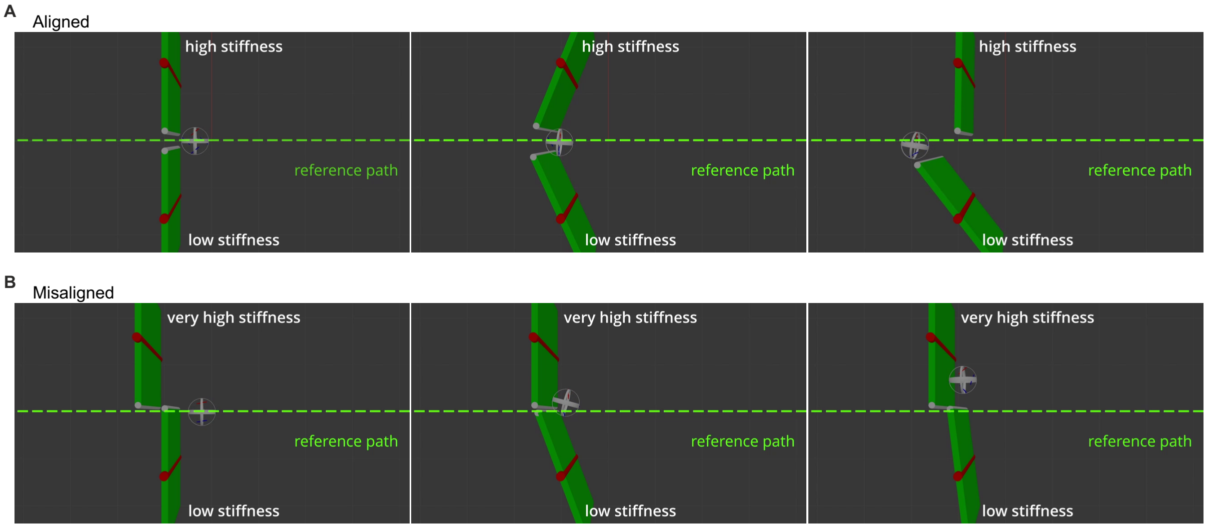

Supplementary Figure 4: **Traversal of multiple obstacles. A** Example of a successful scenario where two obstacles are aligned and equally spaced apart from the reference path. **B** Example of a failure case due to the rigidity of one of the two obstacles and the misalignment with respect to each other.

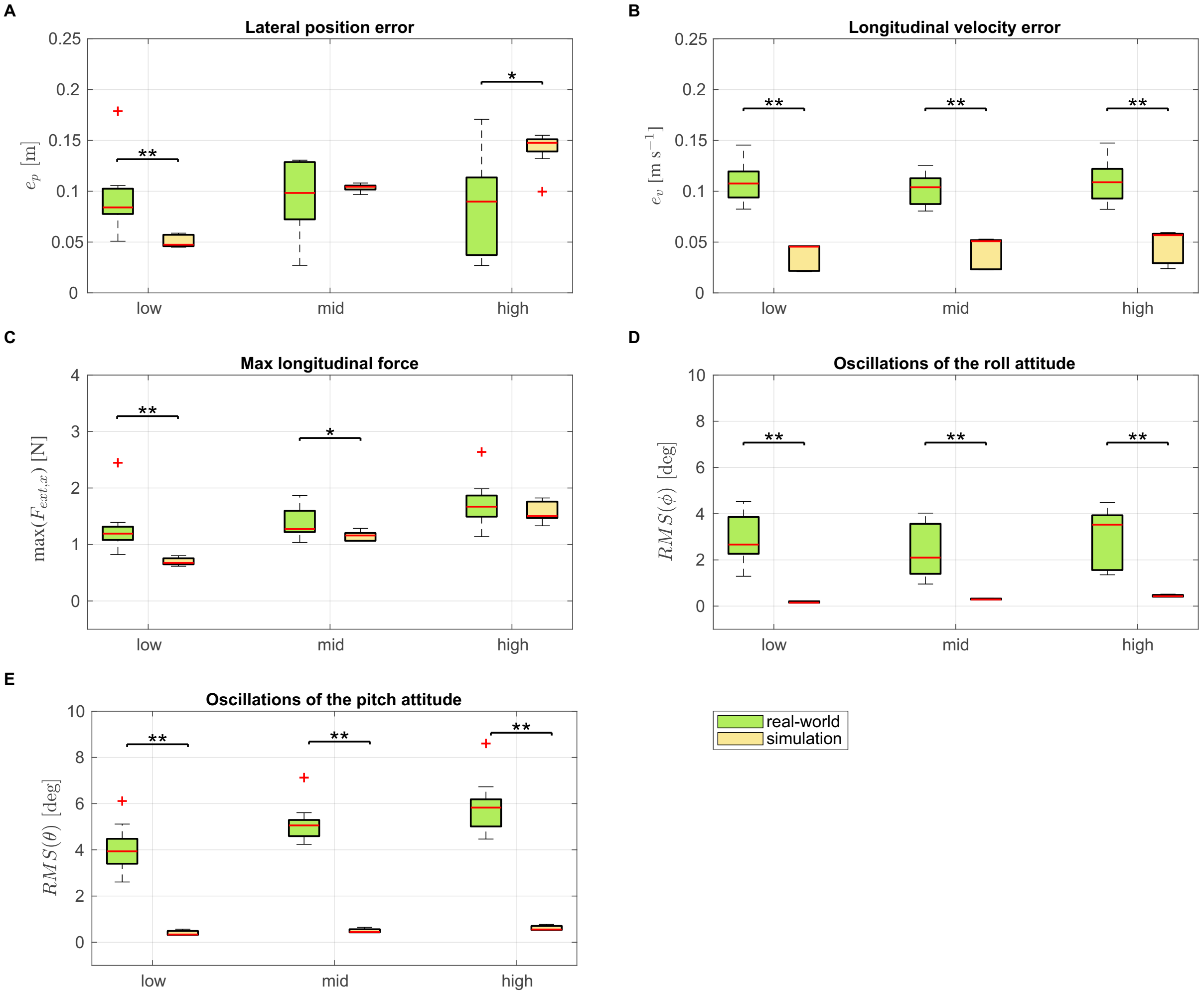

Supplementary Figure 5: **Assessment of the sim-to-real gaps for experiments conducted with balanced weight.** **A** Tracking error of the lateral position; **B** longitudinal velocity error; C max longitudinal force; **C** max longitudinal force; **D** oscillations on the roll motion; **E** oscillations on the pitch motion. Mann-Whitney U test performed among the two sets for all the stiffnesses; box plots indicate median (middle line), 25th, 75th percentile (box) and 5th and 95th percentile (whiskers) as well as outliers (single points); number of tests used for the statistical analysis = 9 for each stiffness; (no asterisk) P > 0.05, * P < 0.05, ** P < 0.01.
