# Supplementary material for: Synergistic morphology and feedback control for traversal of unknown compliant obstacles with aerial robots
Source: Nat Commun. 2024 Mar 26;15:2646. doi: 10.1038/s41467-024-46967-5 (PMC10965961; doi:10.1038/s41467-024-46967-5)
Supplement: Supplementary file 3 — Description of Additional Supplementary Files [file 41467_2024_46967_MOESM3_ESM.pdf]

Description of Additional Supplementary Files

Supplementary Movie S1 - Inspired by animal locomotion, the tight interplay of body morphology and sensory-driven control allows drones to traverse compliant obstacles via physical interaction.

Supplementary Movie S2 - Traversal of compliant hinged plates ranging three values of stiffness over one order of magnitude.

Supplementary Movie S3 - Ablation study with non-streamlined cage, high-friction shell, and control without force feedback.

Supplementary Movie S4 - Traversal of tree branches with and without leaves.

Supplementary Movie S5 - Impact of the controller parameters on the behavior of the drone during traversal.

Supplementary Movie S6 - Preliminary study on the traversal of multiple compliant obstacles.
